# Working Memory Training Effects on White Matter Integrity in Young and Older Adults

**DOI:** 10.3389/fnhum.2021.605213

**Published:** 2021-04-14

**Authors:** Sabine Dziemian, Sarah Appenzeller, Claudia C. von Bastian, Lutz Jäncke, Nicolas Langer

**Affiliations:** ^1^Department of Methods of Plasticity Research, Institute of Psychology, University of Zurich, Zurich, Switzerland; ^2^Department of Psychology, University of Sheffield, Sheffield, United Kingdom; ^3^Neuroscience Institute, University of Sheffield, Sheffield, United Kingdom; ^4^Institute of Psychology, Department of Neuropsychology, University of Zurich, Zurich, Switzerland; ^5^University Research Priority Program “Dynamic of Healthy Aging”, University of Zurich, Zurich, Switzerland; ^6^Center for Reproducible Science, University of Zurich, Zurich, Switzerland; ^7^Neuroscience Center Zurich (ZNZ), Zurich, Switzerland

**Keywords:** working memory training, working memory, healthy aging, diffusion tensor imaging, white matter integrity, tractography

## Abstract

**Objectives:**

Working memory is essential for daily life skills like reading comprehension, reasoning, and problem-solving. Healthy aging of the brain goes along with working memory decline that can affect older people’s independence in everyday life. Interventions in the form of cognitive training are a promising tool for delaying age-related working memory decline, yet the underlying structural plasticity of white matter is hardly studied.

**Methods:**

We conducted a longitudinal diffusion tensor imaging study to investigate the effects of an intensive four-week adaptive working memory training on white matter integrity quantified by global and tract-wise mean diffusivity. We compared diffusivity measures of fiber tracts that are associated with working memory of 32 young and 20 older participants that were randomly assigned to a working memory training group or an active control group.

**Results:**

The behavioral analysis showed an increase in working memory performance after the four-week adaptive working memory training. The neuroanatomical analysis revealed a decrease in mean diffusivity in the working memory training group after the training intervention in the right inferior longitudinal fasciculus for the older adults. There was also a decrease in mean diffusivity in the working memory training group in the right superior longitudinal fasciculus for the older and young participants after the intervention.

**Conclusion:**

This study shows that older people can benefit from working memory training by improving their working memory performance that is also reflected in terms of improved white matter integrity in the superior longitudinal fasciculus and the inferior longitudinal fasciculus, where the first is an essential component of the frontoparietal network known to be essential in working memory.

## Introduction

Working memory (WM) is defined as the ability to maintain and manipulate goal-relevant information in the face of interference (Baddeley, 2002, 2003; Jonides et al., 2008). WM is essential for daily life skills including reading comprehension (de Jonge and de Jong, 1996), reasoning, and problem-solving (Shah and Miyake, 1999; Pickering, 2006). Healthy aging of the brain is associated with WM decline that can affect the capability of older people to live independently. Therefore, research on how to prevent WM decline with aging is highly demanded, especially given the current demographic change the world is facing (World Health Organization, 2015).

While advances of pharmacological studies did not find adequate therapies so far, recent studies aimed at non-pharmacological intervention, such as cognitive training and physical exercise (Lautenschlager et al., 2008; Geda et al., 2010; Pieramico et al., 2014). Cognitive training interventions are a promising tool for delaying age-related cognitive decline (Kelly et al., 2014) or even improving cognitive functions (Mahncke et al., 2006; Wolinsky et al., 2006; Cheng et al., 2012; Anguera et al., 2013). The vast majority of cognitive interventions target WM (Kelly et al., 2014).

However, the neural mechanisms underlying the beneficial effects of WM training on aging brains remain unclear and are subject to debate. Neuroimaging techniques facilitate the investigation of the impact of WM training on aging individuals and the exploration of its mechanisms. Several neuroimaging meta-analyses demonstrated that frontoparietal regions are consistently activated during different WM tasks and modalities (Wager and Smith, 2003; Owen et al., 2005; Rottschy et al., 2012; Salmi et al., 2018; Emch et al., 2019). The frontoparietal network typically consists of the lateral prefrontal cortex (PFC), which is associated with encoding, manipulation, and response selection (D’Esposito et al., 2000), and the posterior parietal cortex (PPC), which is relevant for storing, maintaining, and retrieving information (Jonides et al., 1998; Guerin and Miller, 2011; Nielsen et al., 2017). In addition, modality-dependent higher-level sensory areas are also shown to be essential for WM functions (Jung and Haier, 2007).

Thus, efficient communication between these brain areas appears crucial for WM performance. Evidence from functional neuroimaging has revealed increased functional connectivity between PFC and PPC as WM load increases (Ma et al., 2012; Dima et al., 2014). Moreover, coupling between visual areas and the parietal and inferior temporal regions, known as the ventral and dorsal visual stream (Goodale and Milner, 1992; Hebart and Hesselmann, 2012), have been linked to WM performance (Hampson et al., 2006; Cocchi et al., 2013; Langer et al., 2013; Shen et al., 2015). Various WM training studies in healthy young adults revealed alterations in brain characteristics of the frontoparietal network (Langer et al., 2013; Finc et al., 2020). A recent meta-analysis has further substantiated induced functional brain changes in the frontoparietal network following WM training (Duda and Sweet, 2019).

Considerable evidence indicates that in addition to the transformation of functional brain characteristics, WM training can even lead to underlying structural brain plasticity in gray and white matter. Prior longitudinal studies identified gray matter changes in brain areas of the frontoparietal network after cognitive (including WM) training in healthy young and older adults (Lampit et al., 2015; Jiang et al., 2016; Metzler-Baddeley et al., 2016; Román et al., 2016).

The relevant cortical brain regions of the frontoparietal network are connected through several axonal bundles, which can be investigated with diffusion tensor imaging (DTI). However, the literature on WM training investigating effects on diffusion metrics is rather scarce. A few studies indicate WM training might induce changes to frontoparietal network relevant white matter fiber tracts such as the superior longitudinal fasciculus (SLF) (Burzynska et al., 2011; Salminen et al., 2016), the inferior fronto-occipital fasciculus (IFOF) (Salminen et al., 2016), the inferior longitudinal fasciculus (ILF) (Nagy et al., 2004) and the forceps minor (Takeuchi et al., 2011; Salminen et al., 2016). However, only Salminen included an active control condition, so it is unknown whether previously reported training effects were specific to WM training or if the neural changes would have been seen with any intensive training task, regardless of cognitive domain. Moreover, white matter changes following WM training were only investigated in young participants. Thus, it remains unclear to what extent WM training has the potential to induce white matter plasticity in older adults.

The present paper aims to investigate the effects of WM training on white matter integrity in young and older participants. Hereby, an improvement in white matter integrity denotes potentially beneficial changes in neuronal microstructure (e.g., increased myelination or spatial rearrangement of fibers) that are operationalized as changes in diffusivity measures from DTI scans. For this purpose, we conducted a longitudinal DTI study to investigate the effects of an intensive four-week adaptive WM training intervention on white matter integrity quantified by the diffusivity measure mean diffusivity (MD). We compared MD for young and older adults who were randomly assigned to an adaptive working memory training group (WM) or an age-matched adaptive active control group (AC). The data presented here are part of a larger study in which we investigated WM training effects on cognitive performance (von Bastian et al., 2013a) and resting-state EEG (Langer et al., 2013) in which we found gains in the trained tasks in both young and older adults. In this study, using a subset of the larger study including participants that completed DTI scans, we expected to replicate the behavioral findings of an improvement in WM performance after the WM training for both age groups in the WM group compared to both age groups of the AC group. Based on previous studies, we expect that WM training induces increased white matter integrity in fiber tracts of the frontoparietal network and ventral visual WM stream, including the superior longitudinal fasciculus (SLF) (Vestergaard et al., 2011; Salminen, 2016), the inferior fronto-occipital fasciculus (IFOF) (Salminen, 2016), the inferior longitudinal fasciculus (ILF) (Salminen, 2016) and the corpus callosum (CC) (Nagy et al., 2004; Charlton et al., 2010; Takeuchi et al., 2010).

Consequently, we hypothesize that both age groups show significantly decreased MD values, reflecting increased white matter integrity, in the above white matter tracts after WM training.

## Methods

### Participants

This study focuses on a subset of 52 participants who took part in a larger research project and completed additional neurophysiology recordings and neuroimaging scans. In the larger research project, we investigated the effects of WM training on cognitive outcomes and changes in EEG resting-state activity, which have been published elsewhere (von Bastian et al., 2013a; Langer et al., 2013); data from the brain imaging measures have not been analyzed previously. The sample consisted of 32 young (19 women, 13 men; mean age = 23, SD = 3.34, age range 19 – 36 years) and 20 older participants (8 women, 12 men; mean age = 69, SD = 3.57, age range 65 – 77 years). Each participant was randomly assigned either to a working memory group (WM) (16 young, 10 older) or an active control group (AC) (16 young, 10 older). The corresponding age groups did not differ regarding their demographic variables of age (young: *t*(30) = −1.01, *p* = 0.32; older: *t*(17) = 0.76, *p* = 0.46), gender (χ^2^ = 0.02, *p* = 0.89), education (young: *z* = −0.80, *p* = 0.42; older: *z* = 0.91, *p* = 0.36), their experience in using a computer (young: all *p* > 0.26; older: all *p* > 0.07), and cognitive activity in daily life (young: *t*(30) = 0.77, *p* = 0.45; older: *t*(17) = −0.12, *p* = 0.91). Basic demographics of the groups are listed in Table 1. Descriptive statistics regarding the experience using a computer are shown in Supplementary Tables 1a,b. Further information regarding the assessment of education is given in the Supplementary Section B. All participants were consistently right-handed according to the Annett-Handedness-Questionnaire (Annett, 1970), and highly proficient Swiss German or standard German speakers. Participants reported no history of psychiatric or neurological disease, neuropsychological problems, or medication and drug abuse. All participants gave written informed consent to participate in the study. This study was conducted according to the principles expressed in the Declaration of Helsinki and was approved by the Institutional Review Board of “Kantonale Ethikkommission” (EK: E-80/2008).

**TABLE 1:** Participants demographics as a function of group.

Age group	Young		Older	
Group	WM	AC	WM	AC
Sample size (n)	16	16	10	10
Age (M ± SD)	22.38 ± 2.33	23.56 ± 4.10	69.70 ± 3.89	68.44 ± 3.28
Gender (w : m)	9 : 7	10 : 6	5 : 5	3 : 7
Education (M ± SD)	5 ± 1	5 ± 1	6 ± 2	5 ± 1

*WM, working memory; AC, active control group; w, women; m, men. Education is shown in ranks ranging from 1 (no formal education) to 8 (doctorate).*

### Procedure

All participants were asked to complete 20 sessions of training (approx. 25 – 30 min per session) over a period of four weeks. Participants assigned to the WM group practiced WM tasks. Training for the participants assigned to the AC group comprised tasks that were not expected to have an effect on WM performance but were similarly challenging and motivating. The study was conducted double-blinded, so that neither participants nor experimenters were aware of the group participants were assigned to. Participants were also not informed about the intended effect of the training. An AC group was incorporated into this study design to control for expectancy effects (Oken et al., 2008) and intervention effects (Mahncke et al., 2006), which here would be the adherence to regular training and the completion of computer-based tasks that demand considerable concentration. Each session consisted of three tasks in randomized order with a duration of approximately 10 min per task. All groups started their first session at the same level of difficulty. Consecutive task difficulty (i.e., level) was adapted based on individual performance (see below in the task description for the details on difficulty adaption). The training was executed at home via the open-source software Tatool (von Bastian et al., 2013b) and results of each session were uploaded to a web server to monitor participants’ commitment. Before and after the training period, participants completed a cognitive test battery comprising a series of cognitive tasks, EEG, and, for the subgroup analyzed in the present article, MRI scans (DTI and T1-weighted scans) in the laboratory. The present study only focuses on diffusivity measures from DTI that quantify neuroanatomical microstructures. Cognitive WM training outcomes were analyzed in terms of a partial replication as premise for subsequent analysis of neuroanatomical measures. The full results of the cognitive outcomes are reported in von Bastian et al. (2013a), and resting-state EEG analysis are reported in Langer et al. (2013).

### Training Intervention

#### Working Memory Training Tasks

The WM training was based on the facet model of WM described in Oberauer et al. (2000, 2003). This model was established using a factor-analytic approach, following the framework of facet theory (Guttman, 1954; Canter, 1985) which suggests the differentiation of the WM along two main facets: *cognitive functions* and *content domains* (Oberauer et al., 2000). The function facet can further be divided into three categories: *storage and processing*, *relational integration*, and *supervision*. Storage and processing refer to simultaneously processing and storing information (Salthouse, 1990) and are often assessed with complex span tasks. Relational integration (also referred to as monitoring) is the coordination of information elements into new structures (e.g., Halford et al., 1998). Finally, supervision represents the selective activation of goal-relevant and inhibition of goal-irrelevant information, and is typically measured with task switching paradigms (Oberauer et al., 2000, 2008). The content domains comprise verbal, numerical, and spatial materials. For each of these categories participants in the WM group trained one specific task in each session: a numeric complex span task for storage and processing, a task switching task for supervision, and the tower of fame task for relational integration (Langer et al., 2013; von Bastian et al., 2013a). The tasks were chosen based on the results of a previous study, which examined the effects of training on the three functional categories separately (von Bastian and Oberauer, 2013).

##### Storage and processing: numerical complex span

**FIGURE 1:**
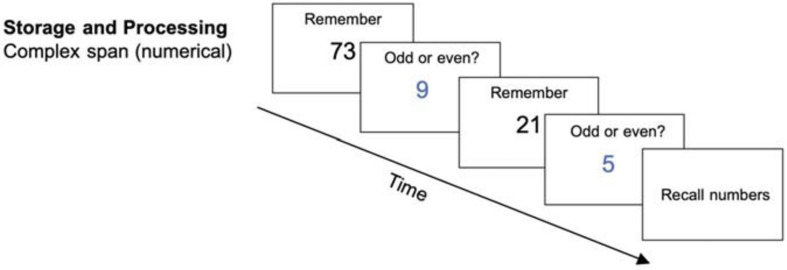
Storage and processing task (complex span) (reused and adapted with permission from Langer et al., 2013). Participants had to memorize the blue digits which were interrupted by a distractor item on which they needed to judge the parity (Langer et al., 2013; von Bastian et al., 2013a).

Each trial started with the central display of a memory item (a two-digit number, printed in black) for 0.5 s. After that, a distractor (a single-digit number, printed in blue) was presented for which participants had to judge the parity (odd or even) as quickly and as accurately as possible within 3 s. The digit remained on the screen until the participant’s response, and a blank screen was shown until the 3 s passed. This was followed by another memory item and then another distractor-decision (see Figure 1). The length of the item-distractor sequence depended on the difficulty level. Afterward, participants had to recall all memory items in the correct order. There was no time limit set for the recall. Each session consisted of 12 recall trials. The achieved level of difficulty was used as a performance measure.

##### Relational integration: tower of fame

**FIGURE 2:**
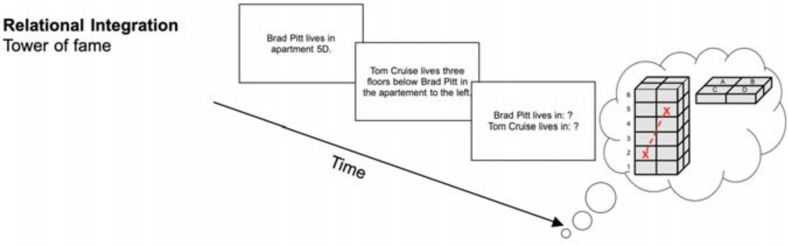
Relational integration task (tower of fame) (reused and adapted with permission from Langer et al., 2013). Participants needed to integrate information into the tower of fame and were then asked about the apartments people lived in Langer et al. (2013); von Bastian et al. (2013a).

The Tower of Fame task (see Figure 2) required participants’ ability to integrate single information elements and the relations between them. Participants had to imagine a tower with six floors, each consisting of four apartments (A, B, C, and D). They were then sequentially presented with statements about the location of famous people’s apartments. Each trial started with a statement about one particular apartment (e.g., “Tom Cruise lives in apartment 2A.”) and was then followed by statements with information relative to the first statement (e.g.,“Brad Pitt lives two floors above Tom Cruise in the apartment to the right.”). At the end of each trial, participants had to recall the apartments for each of the people mentioned (e.g., “Tom Cruise lives in? – 2A” and “Brad Pitt lives in? – 4B”). At increased difficulty levels the order of recall was randomized and more statements were presented. Hence, participants had to memorize the binding between apartment numbers (e.g., “2A”) and names (e.g., “Tom Cruise”). Each session comprised 15 trials. Performance was measured by the achieved level of difficulty.

##### Supervision: figural task switching

**FIGURE 3:**
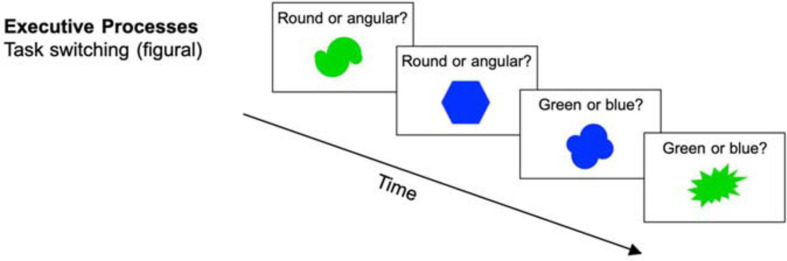
Executive processes task (task switching) (reused and adapted with permission from Langer et al., 2013). Participants were presented with stimuli on which they had to decide if they were round vs. angular or green vs. blue dependent on the current task (Langer et al., 2013; von Bastian et al., 2013a).

In this task, participants were presented with bivalent stimuli (simple geometrical shapes, see Figure 3). These stimuli had to be categorized based on set criteria that switched every two trials. For example, participants had to switch between determining whether a shape had a border and whether it was dotted or striped. The decision had to be made as accurately and as quickly as possible. At increased difficulty levels, display duration, and therefore the time to respond to the stimulus was set to the 99th percentile of the individual reaction times (RT) based on all previous trials since the last adjustment (see von Bastian and Oberauer, 2013). Additionally, in every fifth session, the sets of stimuli were replaced (i.e., new stimuli and new categorization rules) in order to enhance variability. Each session consisted of 384 trials. Performance was measured as the proportional switch cost, which is the reaction time difference between task switch trials and repetition trials divided by the average reaction time of both switch and repetition trials.

#### Active Control Training Tasks

To hold the variability of the training tasks constant, the AC group completed three different tasks in each training session as well. As the WM group, the AC group performed 20 sessions of training at home with approximately the same duration. Their training consisted of a general knowledge quiz, a visual search task, and a counting task (von Bastian et al., 2013a; Langer et al., 2013).

##### Quiz

Participants had to answer questions taxing general knowledge by choosing one of four response options within 60 s. The training consisted of 3’507 questions provided by the Quiz-Fabrik GmbH. Each session comprised 100 trials. Task difficulty was increased according to the item difficulty provided by Quiz-Fabrik. Performance was measured by the percentage of correct answers.

##### Visual search

Participants were presented multiple circles with two gaps and were asked to search for a target item, a circle with only one gap. Participants then had to point out the direction of the gap with the arrow keys within 60 s. In trials without a target, participants had to press “A.” At increased difficulty levels a larger number of circles was displayed. In each session, participants had to complete 70 trials. Performance was measured as the percentage of correct answers. If there was no answer given by the participant in a trial, the response was treated as incorrect.

##### Counting

Blocks of identical digits were presented for 60 s. These blocks followed the rule that they contained as many digits as the digit indicated (e.g., a sequence of three 3s, or a sequence of five 5s). Participants were asked to determine whether any block presented broke this rule, and then press the respective number. If all blocks were correct, participants had to press “0.” Each session comprised 70 trials. The percentage of correct answers was used as the performance score. In the case of not providing a response, the trial was counted as incorrect.

### Pre- and Post-assessment

Before and after the training intervention, participants completed a cognitive test battery comprising the three trained WM tasks, three structurally similar tasks with different materials, three structurally different WM tasks, and one (older adults) or two (young adults) reasoning tasks (for detailed descriptions, see von Bastian et al., 2013a). In a supplementary analysis (see Supplementary Section B) of the behavioral data, that is a partial replication of a previous study (see von Bastian et al., 2013a), we found that the WM training group showed performance gains in all training tasks; however, only for the complex span and the Tower of Fame task, but not for the switching task, were these improvements were significantly different from those observed in the AC group. This supplementary analysis on behavioral training effects served as a prerequisite for the consecutive DTI analysis, demonstrating WM training gains are also present in the subsample for which we had DTI data. In line with the previous larger study (see von Bastian et al., 2013a; Langer et al., 2013), in the present study, we focused exclusively on data from the complex span and the Tower of Fame task. The pretest/posttest versions of these tasks were exactly identical to the training versions, except that the difficulty levels were fixed. The complex span task comprised 15 trials with set sizes ranging from 3-7 numbers, and the Tower of Fame task comprised 18 trials with 2-4 statements and pseudo-randomized order of recall. We computed a composite WM score for pretest and posttest by averaging the z-transformed scores of both tasks as follows:

$$C\,o\,m\,p\,o\,s\,i\,t\,e\,W\,M\,S\,c\,o\,r\,e=\frac{\begin{array}{c} z\,\left(S\,t\,o\,r\,a\,g\,e\,a\,n\,d\,P\,r\,o\,c\,e\,s\,s\,i\,n\,g\right)+\\ z\,\left(R\,e\,l\,a\,t\,i\,o\,n\,a\,l\,I\,n\,t\,e\,g\,r\,a\,t\,i\,o\,n\right) \end{array}}{2}$$

### MRI Data Acquisition

The magnetic resonance imaging (MRI) scans were acquired on a 3.0 T Philips Achieva whole-body scanner (Philips Medical System, Best, The Netherlands) equipped with a transmit-receive body coil and a commercial eight-element sensitivity encoding (SENSE) head coil array. Volumetric 3D T1-weighted gradient-echo sequence scans were obtained with a measured spatial resolution of 0.94 × 0.94 × 1 mm (acquisition matrix 256 x 256 pixel, 160 slices). Further imaging parameters were: field of view FOV = 240 × 240 mm, echo time TE = 3.7 ms, repetition time TR = 8.06 ms, flip angle = 8, and SENSE factor R = 2.1.

Diffusion-weighted spin echo-planar (EPI) sequence scans were obtained with a measured spatial resolution of 2.0 × 2.0 × 2.0 mm (acquisition matrix 112 × 112 pixels, 75 slices). Further imaging parameters were: field of view FOV = 224 × 224 mm, echo time TE = 55 ms, repetition time TR = 13.006 ms, flip angle FA = 90, and SENSE factor *R* = 2.1. Diffusion was measured in 64 non-collinear directions preceded by a non-diffusion-weighted volume (reference volume). The b-value was 1.000 s/mm^2^.

#### Preprocessing

Diffusion-weighted images of all participants and both time points underwent identical processing steps using the FMRIB Software Library (FSL) version 6.0 (Jenkinson et al., 2012). First, the FSL Brain Extraction Tool (BET) was applied, extracting the brain from non-brain tissue from the whole head image resulting in a binary brain mask (Smith, 2002). The brain mask was created with a fractional anisotropy (FA) threshold of 0.2. In the following step, eddy current-induced distortions, which are a common artifact of diffusion images, were removed from the data using the FSL tool “eddy_cuda”. This tool also corrects for artifacts of participant’s in-scanner head motion (Andersson and Sotiropoulos, 2016; Andersson et al., 2016, 2017). In detail, the eddy_cuda tool was applied with the number of iterations set to 8 and decreasing smoothing full-width-half-max parameters (10,6,4,2,0,0,0,0) in each iteration. Outlier detection and replacement were enabled for slice-wise outliers which concern signal dropouts that occur within a single slice. Furthermore, the parameters of eddy_cuda were set to run additional 8 iterations for the slice-to-volume correction, which considers movement within a volume instead of between volumes by modeling 9 degrees of freedom in movement for each volume. Eddy_cuda was chosen over previous eddy versions as it additionally implements intra-volume movement correction and therefore reduces remaining artifacts and its potential impacts on measures extracted further down in the processing pipeline (Andersson et al., 2017).

The subsequent preprocessing steps included diffusion tensor fitting to obtain diffusion measures (e.g., FA and MD) at each voxel by calculating the tensors of the diffusion weights images based on a linear regression using the tool “dtifit” from FMRIB’s Diffusion Toolbox. This step was followed by ac-pc alignment on a T1-weighted image from Talairach coordinates into MNI space by using the function “mrAnatAutoAlignAcpcNifti” from the vista-soft toolbox developed by the Stanford Vista Lab, 2016^1^. The T1-weighted image served as an anatomical reference for subsequent extraction of measures of white matter integrity using the Automated Fiber Tract Quantification (AFQ) toolbox version 1.2 (Yeatman et al., 2012) in MATLAB 2015b.

AFQ is a deterministic tractography algorithm that identifies 20 major fiber tracts and calculates diffusion properties for 100 equidistant nodes of each tract yielding individual fiber tract profiles. Relevant to the present study, tractography was obtained on fiber tracts, which belong to the frontoparietal network and revealed a significant relationship with WM performance in previous publications (Nagy et al., 2004; Charlton et al., 2010; Takeuchi et al., 2010; Vestergaard et al., 2011; Salminen, 2016). Specifically, tractography was conducted for the callosum forceps minor, the left and right IFOF, the left and right SLF, the left and right ILF, and the left corticospinal tract (CST). The latter was chosen as a control tract expecting to have no training-induced changes.

In brief, the AFQ tractography algorithm is implemented as follows: first, a whole-brain tractography was estimated with a deterministic streamline tracking algorithm (STT) (Mori et al., 1999) by seeding voxels with FA values greater than 0.2. The tracking was interrupted when either (1) the FA value at that specific position dropped below 0.25 or (2) the minimal angle between the current and the following segment was greater than 35° implying an unusual fiber orientation. Second, fiber tracts were segmented by a waypoint ROI procedure where all fibers passing through the same two ROIs, as identified by the participant’s T1-weighted scan, were assigned to the identical fiber group (Wakana et al., 2004). Third, each fiber tract was matched with fiber tract probability maps as created by Hua et al. (2008). Fibers exceeding a maximum aberrance of 0.25 were excluded from further analyses. Fourth, fibers deviating more than 4 standard deviations from the mean fiber length or more than 5 standard deviations from the core of the fiber tracts were iteratively eliminated at this point. Fifth, after eliminating all potential outliers, the fiber tracts were clipped at the two waypoint ROIs and then resegmented into 100 equidistant nodes where diffusion properties (i.e., FA and MD) were computed (Yeatman et al., 2012). These properties were computed as weighted averages of the diffusion properties of all fibers within the node, with the weights being the probability that the fibers are part of the fiber group.

DTI allows for the extraction of various microstructural measures based on the diffusion of water molecules that is restricted by impermeable tissue. Water can diffuse best along the direction of myelinated fiber tracts, microtubules, and cell membranes. Therefore, measures of diffusivity can be leveraged to quantify neuroanatomical microstructures. These diffusivity measures include axial diffusivity (AD), radial diffusivity (RD), FA, and MD. AD represents the diffusion rate along the principal axis (Soares et al., 2013; Curran et al., 2016), whereas RD reflects the perpendicular diffusivity to the principal axis, respectively. FA characterizes the restriction of diffusion and is defined as the degree of anisotropy of water diffusion at a given voxel. FA is commonly associated with increased myelination and white matter fiber health although it is strongly influenced by various other factors such as axon branching, packing density, axon diameter, number of axons, and fiber crossing (Zatorre et al., 2012). The latter has often been referred to as the *“crossing fiber problem”* in literature (Alexander and Seunarine, 2010; Tournier, 2010; Jones et al., 2013). Finally, MD is a measure reflecting overall diffusivity in any direction and is defined as the mean of the three eigenvectors of the diffusion tensor (Soares et al., 2013). In a study by Cox et al. (2016), they compared various microstructural diffusion measures on a large sample from the United Kingdom Biobank. They found MD to be most sensitive to identify white matter changes in aging populations. In contrast to FA, MD was non-linearly associated with increased age reflected in a steeper slope and thus stronger differentiation in older age (Cox et al., 2016). In addition, several papers have demonstrated that MD is more sensitive to study short-term plasticity. Sagi et al. (2012) and Tavor et al. (2013) also chose MD as a measure of microstructural fiber organization, to investigate short-term plasticity changes after learning. Their choice of using MD over other measures is based on a previous study by Hofstetter and Assaf (2017) in which rapid MD changes were evident after short periods of training. Therefore in this paper, statistical analysis was conducted using MD as the measure quantifying change in white matter integrity with the focus on older participants.

Here, we investigated tract-wise MD, for the eight above mentioned fiber tracts. For those we extracted MD for each of the 100 nodes between the ROI of the tract using AFQ and presented the descriptive statistics of the individual nodes. For statistical analyses, we calculated the average MD value along these 100 nodes. Hence, we obtained one MD value per tract for the eight previously mentioned tracts of interest. Furthermore, we also investigate MD changes on the whole-brain level, subsequently referred to as global MD.

#### Quality Metrics

Beyond the extraction of measures of interest, DTI data were also analyzed for metrics reflecting imaging quality for both time points separately. We used the “eddy_openmp” tool and the “eddy_quad” (QUality Assessment for DMRI) tool from FSL version 6.0.03 to extract estimates of in-scanner head motion and b-shell-wise CNR metrics (Bastiani et al., 2019). The final CNR score was defined as the average value across all b-shell metrics as reported by eddy_quad (Bastiani et al., 2019). In-scanner head motion was calculated as the average relative motion across all volumes.

Two participants of the older WM group showed a disproportional low CNR for the DTI scan of the first time point (i.e., 5 median standard deviations from the median over all subjects for each specific analysis), for which reason they were excluded from subsequent analyses of neuroanatomical measures. Exclusion of those two participants served to maintain high image quality regarding the small sample sizes of the subgroups and ensure data quality differences are not biasing group results.

### Statistical Analyses

All statistical analyses were conducted using the Statistics and Machine Learning Toolbox version 11.5 in MATLAB 2019a. In the following formulas fixed effects are denoted by a “+” symbol and interaction effects by an “^∗^” symbol in line with the Wilkinson notation (Wilkinson and Rogers, 1973). All effect sizes of significant effects from the generalized linear mixed-effects model analyses were calculated using Cohen’s *f2* (Selya et al., 2012).

For a replication of the behavioral training gains in each task within the subsample used in this study see Supplementary Section B.

#### Behavioral Training Effects

Behavioral training effects were analyzed with a generalized linear mixed-effects model using the *composite WM score* of the pre- and post-assessment as the dependent variable, *age* (young/older), *training* (WM group/AC group) and *time point* (pre-assessment/post-assessment) as the fixed effects and *subject* as the random effect. We also specified random slopes for *age*, *training*, and *time point*, resulting in the following formula used:

composite⁢WM⁢score∼age*training*time⁢point+

(1|subject)+(-1+age|subject)+(-1+training|subject)+

     (-1+timepoint|subject)

#### Effects on In-Scanner Head Motion

For assessing the effects on in-scanner head motion a generalized linear mixed-effects model was used that specified the *head motion* as dependent variable and *age* (young/older), *training* (WM group/AC group) and *time point* (pre-assessment/post-assessment) as fixed effects in addition to a random effect of *subject* and random slopes for *age*, *training*, and *time point*. Therefore, the model was specified as follows:

head⁢motion∼age*training*time⁢point+(1|subject)+

(-1+age|subject)+(-1+training|subject)+

(-1+time⁢point|subject)

#### Effects on CNR

Effects on contrast-to-noise-ratio (CNR) were estimated using a generalized linear mixed-effects model. Hereby, the dependent variable was the CNR value and *age* (young/older), *training* (WM group/AC group), *time point* (pre-assessment/post-assessment) and in-scanner *head motion* were defined as fixed effects and *subject* as random effect. Additionally, there were also random slopes defined for *age*, *training*, *time point*, and in-scanner *head motion* yielding the following model:

CNR∼age*training*time⁢point+head⁢motion+

(1|subject)+(-1+age|subject)+(-1+training|subject)+

(-1+time⁢point|subject)+(-1+head⁢motion|subject)

#### Effects on White Matter Integrity

To investigate the effects of the WM training on measures of MD, generalized linear mixed-effects models were calculated. This was performed for global MD and for the previously mentioned tract-wise MD values. For the subsequent analysis, the following generalized linear mixed-effects model was calculated separately for *global MD* and *tract-wise MD*.

MD∼age*training*time⁢point+CNR+head⁢motion

+(1|subject)+(-1+age|subject)+(-1+training|subject)+

(-1+time⁢point|subject)+(-1+CNR|subject)+

(-1+head⁢motion|subject)

Hereby, the dependent variable *global MD* and *tract-wise MD*, respectively, were estimated by the fixed effect of *age* (young/older), *training* (WM group/AC group), *time point* (pre-assessment/post-assessment), *CNR*, and in-scanner *head motion*, as well as by a random effect of *subject*, while considering random slopes for each of the above fixed effect variables.

To interpret the threefold interactions of *age*, *time point*, and *training*, the same generalized linear mixed-effects model was calculated separately for each age group, thus reducing the model to the following:

MD∼training*time⁢point+CNR+head⁢motion+

(1|subject)+(-1+training|subject)+

(-1+time⁢point|subject)+(-1+CNR|subject)+

(-1+head⁢motion|subject)

## Results

### Pre- and Post-assessment of Working Memory Performance

First, we applied an unpaired *t*-test independently for the young and the older group to review potential pre-assessment differences between the WM and AC group. The results indicated no difference in the composite WM score between the WM group and the AC group at the time of pre-assessment for the young group, *t*(30) = −1.54, *p* = 0.13, and the older group, *t*(18) = −0.22, *p* = 0.83. To investigate the effect of *age, training*, and *time point*, we conducted a generalized linear mixed-effects model analysis as introduced above.

The analysis from pre- to post-assessment (see Table 2) yielded a significant main effects of *age* on the composite WM score, β = 1.13, *p* = 2.481×10^−6^, 95% *CI* = [0.68, 1.58], *f*^2^ = 1.17, indicating higher WM scores for the young participants compared to the older participants. The interaction of *training × time point* yielded significant effects on the composite WM score, β = 0.74, *p* = 0.005, 95% *CI* = [0.23, 1.26], *f*^2^ = 0.10, indicating for both age groups greater increases in WM performance for the WM group than for the AC group. There was no interaction effect of *age*, *training*, and *time point*.

**TABLE 2:** Generalized linear mixed-effects model results for the composite WM score.

Name	Estimate	*p*	95% CI	*f*^2^
age	1.134	2.481 × 10^−6^* **^^	[0.684, 1.584]	1.168
training	0.067	0.809	[−0.481, 0.616]	n/a
time point	0.350	0.060	[−0.015, 0.716]	n/a
age × training	0.282	0.426	[−0.418, 0.981]	n/a
age × time point	0.185	0.432	[−0.281, 0.651]	n/a
training × time point	0.743	0.005*	[0.226, 1.260]	0.104
age × training × time point	0.088	0.792	[−0.571, 0.746]	n/a

*p < 0.05, **p < 0.005, ***p < 0.0005. age (0 = older, 1 = young); training (0 = control, 1 = training); time point (0 = pre-assessment, 1 = post-assessment); df = 96.

### DTI Scan Quality Metrics

To assess the effect of *age*, *training*, and *time point* on the quality metrics of the DTI scans, a generalized linear mixed-effects model was fit with the dependent variable being (1) the averaged relative in-scanner head motion estimate and (2) the average CNR.

#### In-Scanner Head Motion

The model for in-scanner head motion revealed significance for the main effect of *age*, β = 0.10, *p* = 0.03, 95% *CI* = [0.01, 0.18], *f*^2^ = 0.07, indicating increased motion in young participants. There was also a significant interaction effect of *training × time point*, β = −0.17, *p* = 0.01, 95% *CI* = [−0.31, −0.04], *f*^2^ = 0.06, revealing a decrease of in-scanner head motion in the WM group from pre-assessment to post-assessment compared to the AC group. Therefore, in-scanner head motion was used as random effect in all subsequent analyses studying the effects of WM on white matter integrity. Complete model results are included in Supplementary Table 2.

#### Contrast to Noise Ratio (CNR)

For the average CNR the model yielded a significant effect only for the main effect of *age*, β = 0.22, *p* = 9.661×10^−5^, 95% *CI* = [0.11, 0.33], *f*^2^ = 0.33, suggesting greater CNR in young participants. As a consequence, CNR was used as a random effect in all subsequent analyses. Detailed results of this model are provided in Supplementary Table 3.

### Neuroanatomical Results

#### Global Mean Diffusivity

A generalized linear mixed-effects model was used to assess the effect of *age*, *training*, and *time point* on the measure of global MD. The model revealed a significant main effect of *age*, β = −0.12, *p* = 0.001, 95% *CI* = [−0.19, −0.05], *f*^2^ = 0.11, indicating increased MD in the older group. No other effects and interaction effects reached significance. The results for this model are shown in Supplementary Table 4a. When splitting the model into the two age groups there was no effect of concern that reached significance in either of the age groups (see Supplementary Tables 4b–c).

#### Tract-Wise Averaged Mean Diffusivity

A generalized linear mixed-effects model was calculated to assess the effect of *age*, *training* and *time point* on the tract-wise averaged MD for each of the predefined fiber tracts: the callosum forceps minor, the left, and right IFOF, the left and right SLF, the left and right ILF, and the left CST, where the latter served as control tract in which we were not expecting any training-induced changes. Only the models for the right ILF and the right SLF yielded significant results. Therefore, these are presented in more detail below. The complete results for all fiber tracts are shown in Supplementary Tables 5a–h.

##### Right inferior longitudinal fasciculus

The model for the averaged MD of the right ILF revealed a significant interaction effect of *training × time point, β* = −0.12, *p* = 0.047, 95% *CI* = [−0.24, 0.00], *f*^2^ = 0.04, suggesting a stronger decrease in MD from pre- to post-assessment in the WM group compared to the AC group. We also observed a significant interaction effect of *age × training × time point, β* = 0.17, *p* = 0.024, 95% *CI* = [0.02, 0.33], *f*^2^ = 0.05 (see Supplementary Table 5g).

**FIGURE 4:**
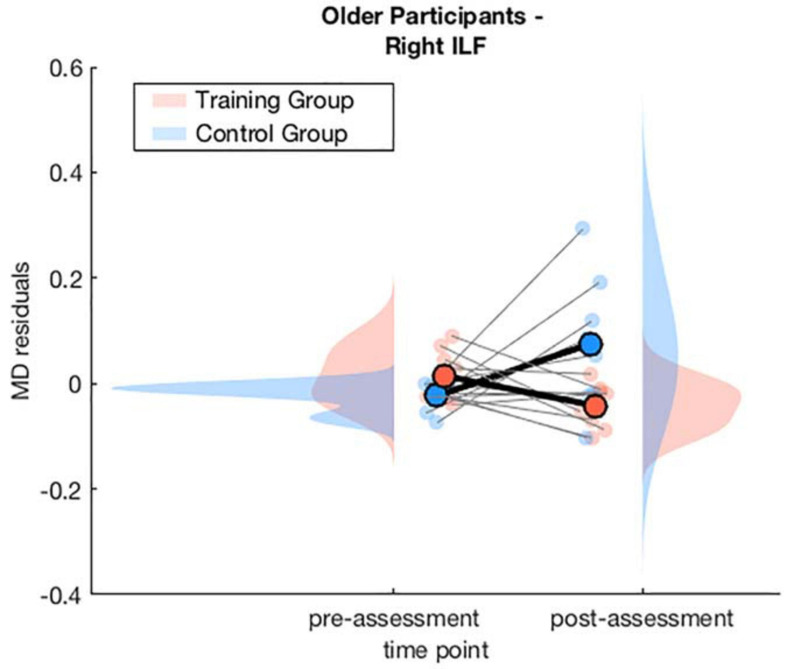
Averaged MD residuals corrected for CNR, in-scanner head motion, and global MD for the right ILF of the WM group and AC group at pre-assessment and post-assessment of the generalized linear mixed-effects model including only older participants.

To interpret the interaction effect of *age × training × time point* on MD changes, separate generalized linear mixed-effects models were calculated disjoint for the young and the older group. For the older group the model for the right ILF revealed a significant interaction effect of *training × time point, β* = −0.14, *p* = 0.028, 95% *CI* = [−0.27, −0.02], *f*^2^ = 0.15, suggesting a decrease of MD in the right ILF in the WM group compared to the AC group after the training intervention (see Table 3 and Figure 4 and for a full report Supplementary Table 7).

**TABLE 3:** Generalized linear mixed-effects model results for average MD in the right ILF for older participants.

Name	Estimate	p	95% CI	f^2^
training	0.058	0.092	[−0.010, 0.126]	n/a
time point	0.102	0.021*	[0.017, 0.188]	0.029
training × time point	−0.141	0.028*	[−0.265, −0.016]	0.149

*p < 0.05, **p < 0.005, ***p < 0.0005. training (0 = control, 1 = training); time point (0 = pre-assessment, 1 = post-assessment); df = 30.

**FIGURE 5:**
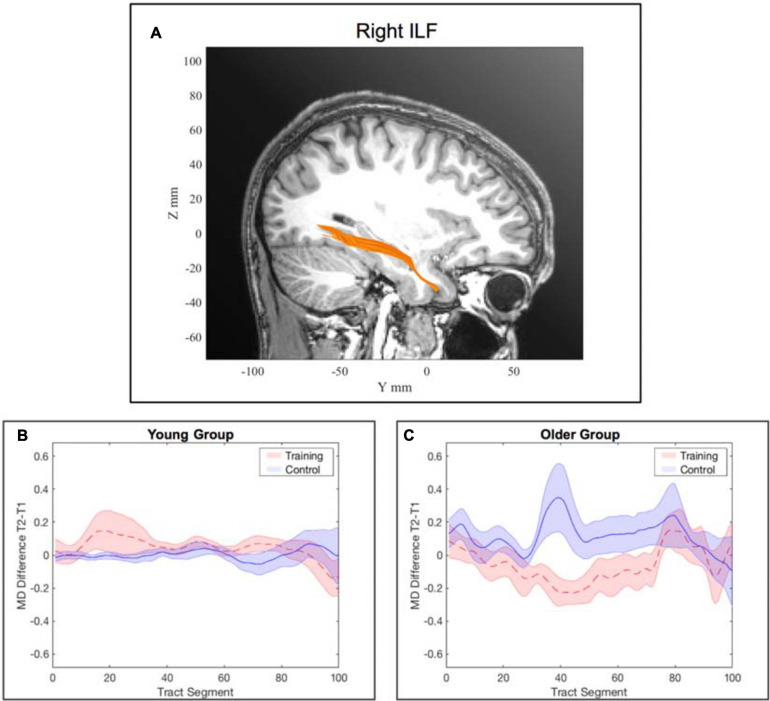
Tract-wise averaged mean diffusivity in the right inferior longitudinal fasciculus (ILF). **(A)** location of the right ILF. **(B)** change in MD from pre- to post-assessment along the tract segments for the young WM group and young AC group separately. **(C)** change in MD from pre- to post-assessment along the tract segments for the older WM group and older AC group separately. Note: the shaded areas represent the standard error, whereas the lines show the mean across all participants within the corresponding group. Subfigure **(B)** was illustrated for completeness although there was no significant interaction effect of *training* and *time point* for the young participants for the right ILF.

There was no significant interaction effect of *training* and *time point* for the young participants in the separate generalized linear mixed-effects model (see Supplementary Figure B2). See Figure 5 for an illustration of a comparison of segment-wise MD changes in the right ILF for the young and the older participants.

##### Right superior longitudinal fasciculus

In the model for the averaged MD of the right SLF, there was a significant interaction effect of *training × time point, β* = −0.14, *p* = 5.094×10^−5^, 95% *CI* = [−0.20, −0.07], *f*^2^ = 0.09, which indicates a decrease of MD in the WM group from pre- to post-assessment. The interaction effect of *age × training* was also significant, β = −0.13, *p* = 0.008, 95% *CI* = [−0.22, −0.03], *f*^2^ = 0.08, suggesting a stronger decrease in MD for the older participants of the WM group. Finally, there was a significant three-way interaction of *age × training × time point*, β = 0.09, *p* = 0.026, 95% *CI* = [0.01, 0.17], *f*^2^ = 0.15 (see Supplementary Table 5e).

**FIGURE 6:**
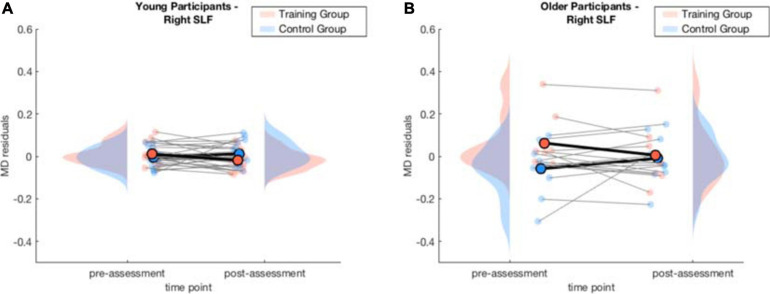
Averaged MD residuals accounting for CNR, in-scanner head motion, and global MD for the right SLF of the WM group and AC group at pre-assessment and post-assessment of the generalized linear mixed-effects model for each age group separately. **(A)** comparison of young WM group and young AC group. **(B)** comparison of older WM group and older AC group.

Again, to interpret the significant three-way interaction, separate generalized linear mixed-effects models were calculated independently for the young and the older group to interpret the effect of *training* and *time point* on MD. The model for the right SLF considering only older participants yielded a significant interaction effect of *time point × training*, β = −0.11, *p* = 0.040, 95% *CI* = [−0.20, −0.01], *f*^2^ = 0.04, revealing that MD decreased in the right SLF in the WM group compared to the AC group after the training intervention (see Table 4 and Figure 6 and for a full report Supplementary Table 8a).

**TABLE 4:** Generalized linear mixed-effects model results for average MD in the right SLF for older participants.

Name	Estimate	p	95% CI	f^2^
training	0.134	0.042*	[0.005, 0.263]	0.095
time point	0.049	0.125	[−0.014, 0.111]	n/a
training × time point	−0.105	0.040*	[−0.204, −0.005]	0.041

*p < 0.05, **p < 0.005, ***p < 0.0005. training (0 = control, 1 = training); time point (0 = pre-assessment, 1 = post-assessment); df = 31.

**FIGURE 7:**
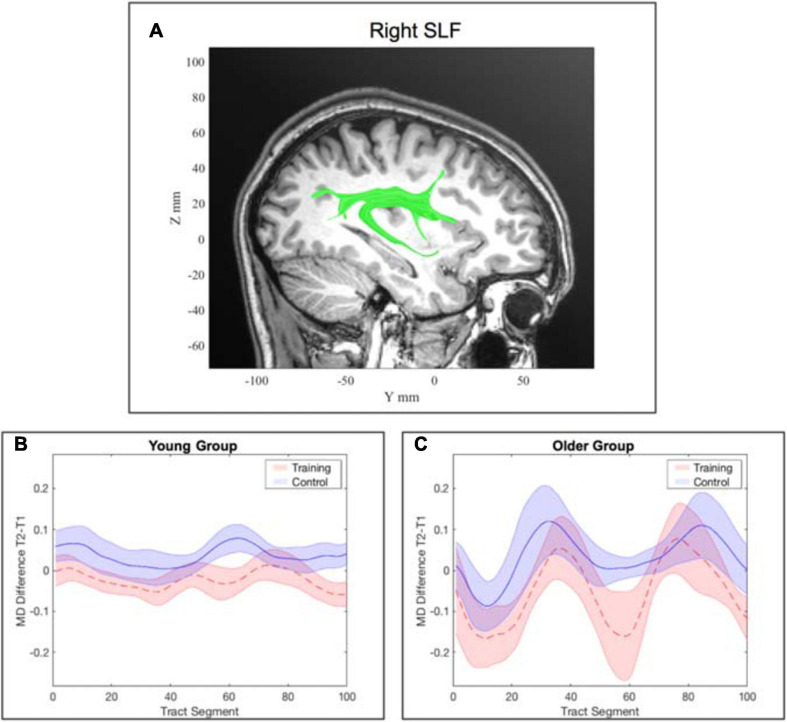
Tract-wise averaged mean diffusivity in the right superior longitudinal fasciculus (SLF). **(A)** location of the right SLF. **(B)** change in MD from pre- to post-assessment along the tract segments for the young WM group and young AC group separately. **(C)** change in MD from pre- to post-assessment along the tract segments for the older WM group and older AC group separately. Note: the shaded areas represent the standard error, whereas the lines show the mean across all participants within the corresponding group.

For the young participants, the model on average MD in the right SLF revealed a significant interaction effect of *training × time point*, β = −0.05, *p* = 0.003, 95% *CI* = [−0.08, −0.02], *f*^2^ = 0.06, showing that MD decreased in the right SLF in the WM group compared to the AC group after the training intervention (see Table 5 and Figure 6 and for a full report Supplementary Table 8b). Figure 7 illustrates a comparison of segment-wise MD changes in the right SLF for the young and the older participants.

**TABLE 5:** Generalized linear mixed-effects model results for average MD in the right SLF for young participants.

Name	Estimate	p	95% CI	f^2^
training	0.012	0.455	[−0.019, 0.042]	n/a
time point	0.013	0.228	[−0.008, 0.035]	n/a
training × time point	−0.047	0.003**	[−0.076, −0.017]	0.063

*p < 0.05, **p < 0.005, ***p < 0.0005. training (0 = control, 1 = training); time point (0 = pre-assessment, 1 = post-assessment); df = 57.

## Discussion

The objective of the present study was to identify the neural mechanisms of white matter changes underlying the benefits of an intensive, double-blinded four-week adaptive WM training in young and older participants. Using DTI, we examined MD in fiber tracts that are associated with WM, such as the SLF, ILF, IFOF and the forceps minor of the corpus callosum (Nagy et al., 2004; Burzynska et al., 2011; Takeuchi et al., 2011; Salminen et al., 2016). During preprocessing, we applied within-volume motion correction and quality control such that throughout the statistical analyses we corrected for these potential confounding variables such as in-scanner head motion and the CNR of the scan.

We could demonstrate that for the young and the older participants the training had a positive effect on white matter integrity reflected by decreased MD in tracts associated with the frontoparietal network. The analysis revealed a decrease in MD in the right ILF in the older WM group. There was also a decrease in MD in the right SLF in both the young and the older WM group. Our control analysis demonstrated that WM training-related white matter integrity changes were specific to these tracts, as the control analysis did not show any changes in the left CST after the WM training.

In the following we will first discuss the behavioral results in more detail, followed by the interpretation of the findings of the white matter changes.

### Behavioral Results

The analysis of overall WM performance included a comparison of test performance at all training levels between pre- and post-assessment (i.e., the composite WM score). The results revealed that also within this subsample of the larger study there was an improvement in overall WM performance reflected by the significant main effect of time. This partial replication is in line with a previous study that examined WM performance on the whole study sample (von Bastian et al., 2013a) and as such served as the premise for the analysis of white matter integrity changes. The results indicated an improvement for both the young and the older group. In particular, the results showed higher WM scores for the young participants in comparison to the older participants by the significant main effect of age. In the additional analysis of training gains (Supplementary Section B), we have also observed an increased training gain in the young group. These findings are not surprising, as age-related cognitive decline also affects WM (Mahncke et al., 2006; Wolinsky et al., 2006; Cheng et al., 2012; Anguera et al., 2013; Kelly et al., 2014) and is the main incentive of this study to which we have demonstrated an impactful intervention. Furthermore, we have also observed an improvement in WM performance in the AC groups. This observation is not surprising, as a possible explanation for an improvement in the composite WM score of the AC group might lie in the general effect of retesting, that has been shown in a recent meta-analysis (Scharfen et al., 2018). More importantly, with the design of an AC and a WM group we could differentiate between the improvement of WM performance in the WM group and the retest effects in the AC group and it further allowed us to distinguish changes that were due to intensive training, regardless of the cognitive domain, to changes that could be attributed specifically to the WM training we designed. Thereby, we observed that WM training indeed resulted in an increased improvement in WM performance compared to the improvement in the AC group, that can only be attributed to the specific WM training tasks. The results showed that the improvement of the WM composite score of the AC group was significantly lower than in the WM group, demonstrated by the significant interaction effect of time point and training. Nonetheless, the improvement on WM performance must be considered domain specific. Although in a previous study we demonstrated that the training in one domain resulted in a near transfer to other modalities of the same task (numeric complex span task and verbal complex span task), in the present study, we have not investigated these transfer effects. Instead we leveraged the result of improved performance after the four-week adaptive WM training as a premise for subsequent analysis of white matter integrity changes.

### White Matter Integrity Results

Diffusion metrics are a frequently used measure to quantify neuroanatomical characteristics of white matter structures. MD is a rotationally invariant diffusivity measure reflecting the average amount of water diffusion or displacement of water molecules in a voxel (Soares et al., 2013). Biological factors like cell membranes, myelin sheath, and microtubules hinder water diffusion. Yet, diffusion is fairly unrestricted along the axons of a fiber tract. Dense packing of cell and axonal membranes decrease MD. Therefore, its value is the lowest in complex tissue (Curran et al., 2016). In previous studies on healthy aging, increase in MD has been also linked to aging (Sexton et al., 2014). In particular, the sensitivity of MD compared to other diffusivity measures in identifying healthy aging-related white matter changes was strongest in advanced age, reflected by a pronounced increase of MD in this population (Cox et al., 2016). Thus, we have focused on MD in the present study. This study is the first to investigate WM training effects in white matter in older adults. We have investigated several fiber tracts that were previously associated with WM performance. Observing a decrease in MD after intensive four-week training is in contrast to the general age-related increase in MD (Cox et al., 2016). Accordingly, the observed WM training-related decrease in MD in tracts that are related to WM might be interpreted as plastic neuroanatomical changes in response to the increased WM demand, counteracting the aging-related increase of MD. We could demonstrate that the WM training-induced white matter integrity changes in the older adults in tracts which are part of the frontoparietal network and the ventral visual WM stream. In detail, after four weeks of intensive adaptive WM training, MD was decreased in the right ILF and the right SLF in the WM group compared to the AC group. In the young participants, we observed white matter integrity changes reflected in a decrease of MD in the right SLF, which is a tract of the frontoparietal network, in the WM group compared to the AC group. In addition, we also inspected the left CST as a control tract in which we did not expect any changes and accordingly did not find any WM training-induced changes in both age groups, as this tract is not directly associated with WM.

#### Inferior Longitudinal Fasciculus (ILF)

We have identified WM training-related changes in the right ILF. The ILF is an associating fiber tract located in the temporal and occipital lobe and is part of the ventral visual WM stream (Latini et al., 2017). It resembles a U-shape that connects the extrastriate visual association areas with the temporal regions involved in transferring visual signals to anterior temporal regions. In more detail, the ILF mostly connects the lingual and the superior occipital gyrus to the middle and superior temporal gyrus (Panesar et al., 2018). As such, the ILF is a crucial component of the ventral visual stream responsible for object recognition identification (Latini et al., 2017). The function of the ILF also comprises attention, integration of visual information in visually guided behavior, as it is the case in scanning and discriminating and ordering visual features, object processing, lexical and semantic processing (Choi et al., 2012; Herbet et al., 2018). These are cognitive components essential for the visuo-spatial WM (Herbet et al., 2018).

Although previous studies linked the ILF with the visuo-spatial WM and visuoconstructive abilities, their samples consisted of adolescents (Krogsrud et al., 2018), young adults (Salminen et al., 2016), or a pathological group of participants with multiple sclerosis (Dineen et al., 2009). The present study is the first DTI study to investigate white matter integrity changes after WM training in older adults and further contributes to previous literature providing evidence that the ILF is a relevant tract of visuo-spatial WM, by demonstrating its plasticity due to WM training. The observed decrease in MD in the ILF in the older participants after the WM training intervention can be interpreted as a plasticity response causing improved communication between visual areas and anterior temporal regions responsible for processing of visual information. The absence of a significant reduction of MD in the right ILF in the AC group allows us to account the observed changes in the WM group to the adaptive WM training.

In fact, the effect was driven by a combination of an MD decrease in the training group and an MD increase in the control group. Since there were no significant baseline differences in MD for both groups (see Supplementary Section B), this observation is due to the interaction effect of *training × time point*. This is in line with previous studies that have demonstrated an age-related increase in MD that is in the normal course of healthy aging (Cox et al., 2016). Whereas the decrease in MD in the training group can be interpreted as being indicative for neural changes that were induced by the WM training and thus potentially indicate a means for counteracting cognitive decline in aging.

Particularly, the relational integration task and the storage and processing task highly require the integration of visual and lexical as well as semantic information. Therefore, it is not surprising to see a decrease in MD and thus improved white matter integrity of the right ILF in the older adults after the training.

#### Superior Longitudinal Fasciculus (SLF)

The SLF is an association fiber tract that mainly projects from the temporal and parietal regions to the frontal cortex. It further receives input from the occipital lobe that it projects to the frontal lobe (Schmahmann et al., 2008). The SLF consists of three components: the dorsal, the major, and the ventral component. In particular, the major and the ventral part are involved in WM. The major component bidirectionally links areas of the prefrontal with areas of the parietal cortex and thus constitutes the main fiber tract of the frontoparietal network, which is considered a main neurocircuit of WM. This connection is necessary for the control of spatial attention and visual perceptions and hence is particularly important for visual WM (Vestergaard et al., 2011).

Our observation of decreased MD after the WM training intervention in the older participants suggests that increased white matter integrity of the right SLF is a consequence of neuronal plasticity providing an improvement in processing and retrieval of spatial information. This is in line with the role of the SFL in visuo-spatial awareness and attention (Schmahmann et al., 2008; Chang et al., 2015). Potentially these changes can be associated with the relational integration task that demands visuo-spatial WM. Information about the location of famous people’s apartments in the Tower of Fame task had to be integrated to recall other people‘s apartment locations. Similarly, to the interpretation in 4.2.1, MD increased in the AC group that can be explained on a physiological basis in the sense of the normal age-related course of cognitive aging (Cox et al., 2016), since there were no statistically significant baseline differences in MD between both groups at the time point of pre-assessment (see Supplementary Section B).

There is a substantial body of literature linking the SLF to various facets of WM. In line with our results, Salminen et al. (2016) found increased white matter integrity in the SLF to be related to WM training, yet their study focused on young participants. Another example is a previous lesion study, which identified the SLF to be correlated with spatial WM deficits (Kinoshita et al., 2016). In addition, a study by Burzynska et al. (2011) on young adults also identified that greater WM performance is connected to higher white matter integrity in the SLF. However, the present study is the first to demonstrate white matter integrity changes in the SLF after adaptive WM training in older adults.

Similarly to the ILF, also for this tract, we can disentangle the general learning effects from the effects which are attributed to the WM training as there was no significant decrease in MD in the AC group.

### Limitations and Future Directions

As with all diffusivity measures derived from DTI (i.e., FA, AD, and RD), MD comes with the risk of incorrectly quantifying diffusion in voxels that contain more than one dominant orientation of a fiber (e.g., crossing or bending fibers). Whilst a voxel-wise change in MD cannot be directly linked to the specific type of change in neuronal microstructure (e.g., increased myelination or spatial rearrangement of fibers), MD as a whole is still suitable to identify any change of such kind. Although we cannot directly infer increased myelination or denser packing of membranes from a decreased MD value, we can infer neuroanatomical changes improving the magnitude of water diffusion along a defined direction, such as the direction of a tract. A decrease in MD in association with an improvement in WM performance is likely to be the effect of beneficial microstructural changes in terms of increased white matter integrity.

A major limitation of this study is the small number of participants in each of the four groups (older/young vs. training/control). Although the overall sample size of 52 participants is comparable to previous neuroimaging studies on WM training effects (Burzynska et al., 2011; Takeuchi et al., 2011; Lampit et al., 2015; Jiang et al., 2016; Metzler-Baddeley et al., 2016; Román et al., 2016; Salminen et al., 2016), when being split into the groups, each group size is rather small. However, the small group size is a consequence of the study design that included two age groups and an active control group in both age groups in a longitudinal design. In the future, larger studies are required to confirm these results before the findings can be generalized beyond the context of this study. With additional studies including larger sample sizes cumulatively generalizable outcomes can be derived to design clinical interventions for maintaining or enhancing cognitive capabilities in older people and improving their quality of life.

Furthermore, whilst these results demonstrated that healthy, older people’s improvements in WM tasks (see also previous study von Bastian et al. (2013a)) manifest in neuroanatomical changes in fiber tracts, we did not investigate the long-term continuity of the behavioral and neuroanatomical WM-related changes. Thus, it remains unclear how long these changes will persist and to what extent they can be preserved with or without a continuation of training. Similarly, this study was not designed to assess the course of improvement and determine at which point gains would reach a plateau. Therefore, future research may focus on the optimal amount of training and follow-up training for sustainable and efficient WM improvement. It may also consider DTI scans during the course of learning to gain more detailed insights into the trajectory of WM improvements and its underlying neuroanatomical changes. Finally, it is yet unknown how changes in fiber tracts translate into everyday life functioning, such as in accomplishing daily activities. Future research would benefit from investigating whether WM training can help older people to live independent lives for longer.

## Conclusion

This study is one of a few that longitudinally examined white matter plasticity using diffusion metrics to quantify the structural change in response to a WM training intervention. Although there are several studies that investigated WM training-induced white matter changes, these only included young participants. The present paper offered the first insight into neuroanatomical effects of WM training in older adults.

In this project, we investigated white matter integrity changes using DTI after four weeks of adaptive WM training in older participants. The results of the study first show that an adaptive WM training lead to an improved WM performance. In addition, this study confirms the hypothesis that components of the frontoparietal network, including the dorsal (SLF) and ventral (ILF) visual WM pathway, underwent structural change after WM training. Thus, in line with previous studies, the present findings suggest that these fiber tracts are essential for WM. Incorporating an AC group into the study design allowed for increasing the explanatory power of the observed white matter integrity changes. In particular, this allowed for differentiating changes that were due to intensive training, regardless of the cognitive domain, to changes that were attributed to WM training, thereby increasing the explanatory power of the observed white matter integrity changes.

The present results support the idea that age-related cognitive decline can be counteracted or partially reversed by adequate training that provokes white matter integrity improvements on the basis of neural plasticity. This outcome helps understanding the potential of neural plasticity in older adults. Although healthy aging is supposed to be unavoidably associated with cognitive decline, this study’s evidence of efficacious neural plasticity in old age can provide a basis that cumulatively with additional studies can be used to develop clinical interventions and age-appropriate therapy that is potentially enhancing end prolonging cognitive abilities.

## Data Availability Statement

The datasets presented in this article are not readily available because we do not have permission from the participants to share the raw data. We can only share derivatives of the data. Requests to access the datasets should be directed to NL, n.langer@psychologie.uzh.ch.

## Ethics Statement

The studies involving human participants were reviewed and approved by Institutional Review Board of “Kantonale Ethikkommission” (EK: E-80/2008). The participants provided their written informed consent to participate in this study.

## Author Contributions

SD did the validation, formal analysis, data curation, writing original draft, writing—review and editing, visualisation, and supervision. SA did the validation, formal analysis, data curation, writing original draft, writing—review and editing, and visualization. CB did the conceptualization, methodology, software, investigation, writing—review and editing, resources, project administration, and funding acquisition. LJ did the conceptualization, resources, project administration, and funding acquisition. NL did the conceptualization, methodology, software, validation, formal analysis, investigation, resources, writing—review and editing, supervision, and project administration. All authors contributed to the article and approved the submitted version.

## Conflict of Interest

The authors declare that the research was conducted in the absence of any commercial or financial relationships that could be construed as a potential conflict of interest.

## Footnotes

1 https://github.com/vistalab/vistasoft
