# Supplementary material for: Working Memory Training Effects on White Matter Integrity in Young and Older Adults
Source: Front Hum Neurosci. 2021 Apr 14;15:605213. doi: 10.3389/fnhum.2021.605213 (PMC8079651; doi:10.3389/fnhum.2021.605213)
Supplement: Supplementary file 1 [file Table_1.docx]

Supplementary Material

# A: Tables

**Supplementary Table 1a.** Unpaired t-test for experience using a computer in the young participants.

| Name | t | df | p |
| --- | --- | --- | --- |
| Average Usage Time | 0.620 | 30 | 0.540 |
| Confidence in Usage | 1.148 | 30 | 0.260 |
| Frequency of Usage | 1.054 | 30 | 0.300 |
| Time since Usage | 1.000 | 30 | 0.325 |
| General Experience | -0.070 | 29 | 0.293 |

**Supplementary Table 1b.** Unpaired t-test for experience using a computer in the older participants.

| Name | t | df | p |
| --- | --- | --- | --- |
| Average Usage Time | -1.936 | 17 | 0.070 |
| Confidence in Usage | -0.158 | 17 | 0.877 |
| Frequency of Usage | -1.503 | 17 | 0.151 |
| Time Since Usage | 0.075 | 17 | 0.941 |
| General Experience | -0.458 | 18 | 0.653 |

**Supplementary Table 2.** Generalized linear mixed effects model for in-scanner head motion, df = 96.

| Name | Estimate | p | 95% CI | f^2^ |
| --- | --- | --- | --- | --- |
| age | 0.097 | 0.026 | [0.012, 0.182] | 0.072 |
| training | 0.088 | 0.069 | [-0.007, 0.182] | n/a |
| time point | 0.077 | 0.114 | [-0.019, 0.172] | n/a |
| age × training | -0.074 | 0.225 | [-0.194, 0.046] | n/a |
| age × time point | -0.081 | 0.191 | [-0.203, 0.041] | n/a |
| training × time point | -0.171 | 0.014 | [-0.306, -0.036] | 0.061 |
| age × training × time point | 0.164 | 0.062 | [-0.009, 0.336] | n/a |

**Supplementary Table 3.** Generalized linear mixed effects model for Contrast to Noise Ratio (CNR), df = 93.

| Name | Estimate | p | 95% CI | f^2^ |
| --- | --- | --- | --- | --- |
| age | 0.223 | <0.0005 | [0.114, 0.331] | 0.326 |
| training | 0.100 | 0.091 | [-0.016, 0.217] | n/a |
| time point | -0.024 | 0.585 | [-0.112, 0.063] | n/a |
| head motion | -0.034 | 0.767 | [-0.258, 0.191] | n/a |
| age × training | -0.149 | 0.065 | [-0.308, 0.009] | n/a |
| age × time point | 0.075 | 0.182 | [-0.036, 0.186] | n/a |
| training × time point | 0.022 | 0.737 | [-0.109, 0.153] | n/a |
| age × training × time point | 0.003 | 0.973 | [-0.160, 0.165] | n/a |

**Supplementary Table 4a.** Generalized linear mixed effects model for global mean diffusivity, df = 92.

| Name | Estimate | p | 95% CI | f^2^ |
| --- | --- | --- | --- | --- |
| age | -0.121 | 0.001 | [-0.192, -0.050] | 0.107 |
| training | -0.070 | 0.081 | [-0.147, 0.009] | n/a |
| time point | -0.032 | 0.388 | [-0.104, 0.041] | n/a |
| head motion | -0.075 | 0.323 | [-0.226, 0.075] | n/a |
| CNR | -0.057 | 0.322 | [-0.170, 0.057] | n/a |
| age × training | 0.081 | 0.100 | [-0.016, 0.178] | n/a |
| age × time point | 0.058 | 0.213 | [-0.034, 0.150] | n/a |
| training × time point | 0.060 | 0.271 | [-0.047, 0.166] | n/a |
| age × training × time point | -0.079 | 0.242 | [-0.212, 0.054] | n/a |

**Supplementary Table 4b.** Generalized linear mixed effects model for global mean diffusivity for older participants only, df = 32.

| Name | Estimate | p | 95% CI | f^2^ |
| --- | --- | --- | --- | --- |
| training | -0.076 | 0.131 | [-0.177, 0.024] | n/a |
| time point | -0.051 | 0.307 | [-0.151, 0.049] | n/a |
| head motion | 0.153 | 0.407 | [-0.218, 0.525] | n/a |
| CNR | -0.165 | 0.189 | [-0.417, 0.086] | n/a |
| training × time point | 0.100 | 0.190 | [-0.052, 0.252] | n/a |

**Supplementary Table 4c.** Generalized linear mixed effects model for global mean diffusivity for young prticipants only, df = 58.

| Name | Estimate | p | 95% CI | f^2^ |
| --- | --- | --- | --- | --- |
| training | 0.015 | 0.499 | [-0.029, 0.059] | n/a |
| time point | 0.024 | 0.248 | [-0.017, 0.064] | n/a |
| head motion | -0.151 | 0.023 | [-0.281, -0.021] | 0.099 |
| CNR | 0.000 | 0.100 | [-0.104, 0.104] | n/a |
| training × time point | -0.021 | 0.462 | [-0.078, 0.036] | n/a |

**Supplementary Table 5a.** Generalized linear mixed effects model for the callosum forceps minor, df = 93.

| Name | Estimate | p | 95% CI | f^2^ |
| --- | --- | --- | --- | --- |
| age | 0.009 | 0.857 | [-0.086, 0.104] | n/a |
| training | 0.040 | 0.4167 | [-0.057, 0.137] | n/a |
| time point | 0.006 | 0.876 | [-0.069, 0.080] | n/a |
| global MD | 0.568 | <0.005 | [0.333, 0.803] | 0.295 |
| head motion | -0.080 | 0.367 | [-0.257, 0.096] | n/a |
| CNR | -0.027 | 0.627 | [-0.135, 0.081] | n/a |
| age × training | -0.053 | 0.391 | [-0.176, 0.070] | n/a |
| age × time point | -0.027 | 0.570 | [-0.123, 0.068] | n/a |
| training × time point | -0.047 | 0.395 | [-0.157, 0.063] | n/a |
| age × training × time point | 0.077 | 0.267 | [-0.060, 0.214] | n/a |

**Supplementary Table 5b.** Generalized linear mixed effects model for left inferior fronto-occipital fasciculus (IFOF), df = 89.

| Name | Estimate | p | 95% CI | f^2^ |
| --- | --- | --- | --- | --- |
| age | 0.002 | 0.957 | [-0.072, 0.076] | n/a |
| training | 0.053 | 0.176 | [-0.024, 0.130] | n/a |
| time point | -0.019 | 0.568 | [-0.085, 0.047] | n/a |
| global MD | 0.558 | <0.005 | [0.366, 0.749] | 0.340 |
| head motion | 0.029 | 0.702 | [-0.122, 0.180] | n/a |
| CNR | 0.098 | 0.034 | [0.007, 0.189] | 0.051 |
| age × training | -0.078 | 0.114 | [-0.176, 0.019] | n/a |
| age × time point | 0.020 | 0.632 | [-0.064, 0.105] | n/a |
| training × time point | -0.058 | 0.2521 | [-0.159, 0.042] | n/a |
| age × training × time point | 0.052 | 0.404 | [-0.072, 0.177] | n/a |

**Supplementary Table 5c.** Generalized linear mixed effects model for right inferior fronto-occipital fasciculus (IFOF), df = 88.

| Name | Estimate | p | 95% CI | f^2^ |
| --- | --- | --- | --- | --- |
| age | -0.020 | 0.479 | [-0.078, 0.037] | n/a |
| training | 0.030 | 0.296 | [-0.027, 0.087] | n/a |
| time point | 0.018 | 0.548 | [-0.042, 0.078] | n/a |
| global MD | 0.537 | <0.005 | [0.356, 0.717] | 0.274 |
| head motion | -0.039 | 0.534 | [-0.161, 0.084] | n/a |
| CNR | -0.026 | 0.422 | [-0.089, 0.038] | n/a |
| age × training | -0.050 | 0.167 | [-0.122, 0.021] | n/a |
| age × time point | -0.022 | 0.557 | [-0.096, 0.052] | n/a |
| training × time point | -0.042 | 0.319 | [-0.126, 0.042] | n/a |
| age × training × time point | 0.053 | 0.312 | [-0.051, 0.157] | n/a |

**Supplementary Table 5d.** Generalized linear mixed effects model for left superior longitudinal fasciculus (SLF), df = 88.

| Name | Estimate | p | 95% CI | f^2^ |
| --- | --- | --- | --- | --- |
| age | -0.008 | 0.860 | [-0.092, 0.077] | n/a |
| training | 0.020 | 0.671 | [-0.073, 0.113] | n/a |
| time point | 0.045 | 0.342 | [-0.049, 0.139] | n/a |
| global MD | 0.460 | 0.002 | [0.180, 0.741] | 0.076 |
| head motion | -0.163 | 0.083 | [-0.348, 0.022] | n/a |
| CNR | 0.111 | 0.046 | [0.002, 0.220] | 0.020 |
| age × training | -0.016 | 0.783 | [-0.132, 0.100] | n/a |
| age × time point | -0.048 | 0.408 | [-0.163, 0.067] | n/a |
| training × time point | 0.024 | 0.711 | [-0.105, 0.153] | n/a |
| age × training × time point | -0.037 | 0.645 | [-0.197, 0.122] | n/a |

**Supplementary Table 5e.** Generalized linear mixed effects model for right superior longitudinal fasciculus (SLF), df = 92.

| Name | Estimate | p | 95% CI | f^2^ |
| --- | --- | --- | --- | --- |
| age | 0.011 | 0.735 | [-0.055, 0.077] | n/a |
| training | 0.141 | <0.005 | [0.068, 0.215] | 0.013 |
| time point | 0.058 | 0.009 | [ 0.015, 0.101] | 0.001 |
| global MD | 0.598 | <0.005 | [0.447, 0.749] | 0.445 |
| head motion | -0.043 | 0.442 | [-0.154, 0.068] | n/a |
| CNR | -0.015 | 0.686 | [-0.091, 0.060] | n/a |
| age × training | -0.127 | 0.008 | [-0.220, -0.034] | 0.078 |
| age × time point | -0.035 | 0.210 | [-0.090, 0.020] | n/a |
| training × time point | -0.139 | <0.0005 | [-0.204, -0.074] | 0.088 |
| age × training × time point | 0.091 | 0.026 | [0.011, 0.172] | 0.146 |

**Supplementary Table 5f.** Generalized linear mixed effects model for left inferior longitudinal fasciculus (ILF), df = 91.

| Name | Estimate | p | 95% CI | f^2^ |
| --- | --- | --- | --- | --- |
| age | 0.079 | 0.030 | [0.008, 0.151] | 0.051 |
| training | 0.029 | 0.426 | [-0.043, 0.101] | n/a |
| time point | 0.044 | 0.199 | [-0.023, 0.111] | n/a |
| global MD | 0.4394 | <0.005 | [0.251, 0.627] | 0.221 |
| head motion | -0.028 | 0.711 | [-0.180, 0.123] | n/a |
| CNR | 0.047 | 0.268 | [-0.037, 0.130] | n/a |
| age × training | -0.048 | 0.303 | [-0.141, 0.044] | n/a |
| age × time point | -0.025 | 0.564 | [-0.111, 0.061] | n/a |
| training × time point | -0.034 | 0.492 | [-0.133, 0.064] | n/a |
| age × training × time point | 0.024 | 0.697 | [-0.099, 0.147] | n/a |

**Supplementary Table 5g.** Generalized linear mixed effects model for right inferior longitudinal fasciculus (ILF), df = 91.

| Name | Estimate | p | 95% CI | f^2^ |
| --- | --- | --- | --- | --- |
| age | -0.010 | 0.732 | [-0.066, 0.046] | n/a |
| training | 0.047 | 0.099 | [-0.009, 0.103] | n/a |
| time point | 0.093 | 0.034 | [0.007, 0.178] | 0.017 |
| global MD | 0.412 | <0.005 | [0.232, 0.592] | 0.130 |
| head motion | -0.052 | 0.447 | [-0.186, 0.082] | n/a |
| CNR | -0.041 | 0.314 | [-0.122, 0.040] | n/a |
| age × training | -0.054 | 0.124 | [-0.124, 0.015] | n/a |
| age × time point | -0.098 | 0.073 | [-0.206, 0.009] | n/a |
| training × time point | -0.122 | 0.047 | [-0.243, -0.002] | 0.043 |
| age × training × time point | 0.174 | 0.024 | [0.023, 0.325] | 0.047 |

**Supplementary Table 5h.** Generalized linear mixed effects model for left corticospinal tract (CST), df = 92.

| Name | Estimate | p | 95% CI | f^2^ |
| --- | --- | --- | --- | --- |
| age | 0.130 | 0.047 | [0.002, 0.257] | 0.152 |
| training | 0.059 | 0.371 | [-0.071, 0.189] | n/a |
| time point | -0.001 | 0.982 | [-0.117, 0.115] | n/a |
| global MD | 1.439 | <0.005 | [1.094, 1.783] | 0.608 |
| head motion | 0.178 | 0.160 | [-0.071, 0.426] | n/a |
| CNR | -0.015 | 0.839 | [-0.163, 0.132] | n/a |
| age × training | -0.017 | 0.835 | [-0.181, 0.146] | n/a |
| age × time point | 0.058 | 0.430 | [-0.088, 0.205] | n/a |
| training × time point | -0.062 | 0.466 | [-0.229, 0.105] | n/a |
| age × training × time point | -0.012 | 0.906 | [-0.220, 0.194] | n/a |

**Supplementary Table 6a.** Generalized linear mixed effects model results for the numerical complex span (storage and processing) task, df = 48.

| Name | Estimate | p | 95% CI | f^2^ |
| --- | --- | --- | --- | --- |
| age | 0.200 | 0.051 | [-0.001, 0.401] | n/a |
| time point | 1.700 | 0.131 | [-0.522, 3.922] | n/a |
| age × time point | 4.613 | 0.002 | [1.780, 7.445] | 0.205 |

**Supplementary Table 6b.** Generalized linear mixed effects model results for the tower of fame (relational integration) task, df = 48.

| Name | Estimate | p | 95% CI | f^2^ |
| --- | --- | --- | --- | --- |
| age | 0.388 | 0.042 | [0.014, 0.761] | 0.236 |
| time point | 0.800 | 0.272 | [-0.648, 2.248] | n/a |
| age × time point | 2.700 | 0.005 | [0.855, 4.545] | 0.166 |

**Supplementary Table 6c.** Generalized linear mixed effects model results for the figural task switching (supervision) task, df = 48.

| Name | Estimate | p | 95% CI | f^2^ |
| --- | --- | --- | --- | --- |
| age | 0.006 | 0.779 | [-0.036, 0.047] | n/a |
| time point | -0.027 | 0.184 | [-0.067, 0.013] | n/a |
| age × time point | 0.012 | 0.633 | [-0.039, 0.064] | n/a |

**Supplementary Table 7.** Generalized linear mixed-effects model results for average MD in the right ILF for older participants, df = 30.

| Name | Estimate | p | 95% CI | f^2^ |
| --- | --- | --- | --- | --- |
| training | 0.058 | 0.092 | [-0.010, 0.126] | n/a |
| time point | 0.102 | 0.021 | [0.017, 0.188] | 0.029 |
| global MD | 0.456 | <0.0005 | [0.218, 0.694] | 0.415 |
| head motion | -0.157 | 0.246 | [-0.427, 0.114] | n/a |
| CNR | -0.046 | 0.614 | [-0.233, 0.140] | n/a |
| training × time point | -0.141 | 0.028 | [-0.265, -0.016] | 0.149 |

**Supplementary Table 8a.** Generalized linear mixed-effects model results for average MD in the right SLF for older participants, df = 31.

| Name | Estimate | p | 95% CI | f^2^ |
| --- | --- | --- | --- | --- |
| training | 0.134 | 0.042 | [0.005, 0.263] | 0.095 |
| time point | 0.049 | 0.125 | [-0.014, 0.111] | n/a |
| global MD | 0.587 | < 0.0005 | [0.324, 0.851] | 0.375 |
| head motion | 0.075 | 0.643 | [-0.251, 0.401] | n/a |
| CNR | -0.003 | 0.982 | [-0.256, 0.251] | n/a |
| training × time point | -0.105 | 0.040 | [-0.204, -0.005] | 0.041 |

**Supplementary Table 8b.** Generalized linear mixed-effects model results for average MD in the right SLF for young participants, df = 57.

| Name | Estimate | p | 95% CI | f^2^ |
| --- | --- | --- | --- | --- |
| training | 0.012 | 0.455 | [-0.019, 0.042] | n/a |
| time point | 0.013 | 0.228 | [-0.008, 0.035] | n/a |
| global MD | 0.609 | <0.0005 | [0.459, 0.758] | 0.757 |
| head motion | -0.069 | 0.111 | [-0.154, 0.016] | n/a |
| CNR | 0.084 | 0.032 | [0.008, 0.161] | 0.039 |
| training × time point | -0.047 | 0.003 | [-0.076, -0.017] | 0.063 |

# B: Methods and Results

**Methods: Assessment of Education**

Education was assessed as the highest completed education categorized into 8 levels with the level 0 being “no formal education” and the level 8 being “doctorate level”. Statistical analysis for group differences was performed using the two-sided Wilcoxon rank sum test for equal medians. The corresponding age groups did not differ regarding education (young: *z* = -0.80, *p* = 0.42; older: *z* = 0.91, *p* = 0.36).

**Methods: Training Gains Effects**

Training gains of the WM training intervention were analyzed using a generalized linear mixed-effects model on the subsample of participants that were in the WM group. The performance of the last session and the performance of the first session for each task were predicted by the variables of *age* (young/older) and *time* *point* (pre-assessment/post-assessment), by a random effect of *subject* and random slopes for *age* and *time point*. Thus, the following formula was used:

task performance ~ age * time point + (1|subject) + (-1+age|subject) + (-1+timepoint|subject)

If there was a significant interaction effect of *age* and *time point* a paired t-test, separately for both age groups, was conducted to compare the highest performance of the last session with the highest performance of the first session for each task separately.

**Results: Training Gain Effects**

The generalized linear mixed-effects model revealed a significant interaction effect of *age* × *time* *point* for the numerical complex span (storage and processing) task, *β* = 4.61, *p* < 0.005, 95% *CI* = [1.78, 7.45], *f^2^* = 0.20, suggesting a positive training gain with a stronger effect for the young participants. Similarly, for the tower of fame (relational integration) task, the model yielded a significant interaction effect of *age* × *time point*, *β* = 2.70, *p* = 0.005, 95% *CI* = [0.85, 4.55], *f^2^* = 0.17, also suggesting a positive training gain with a stronger effect in the young participants. For the tower of fame task there was also a significant main effect of *age* in this model, *β* = 0.39, *p* = 0.04, 95% *CI* = [0.01, 0.76], *f^2^* = 0.24, which indicates general greater task performance in young participants. In the model for the figural task switching (supervision) task no effect reached significance. For a full description of the results see Supplementary Table 6a-c.

To disentangle the interaction effects, we applied a paired t-test for all tasks for reasons of completeness including Cohen’s d as an estimate of effect size. The results for the training gain using the paired t-test showed, that for the young WM group the performance of the last session was significantly increased compared to the performance of the first session in the numerical complex span task (storage and processing), *t*(15)= -5.73, *p* < 0.001, *d* = -1.43, and the tower of fame task (relational integration), *t*(15) = -4.79, *p* < 0.001, *d* = -1.20, but not in the figural task switching task (supervision), *t*(15) = 0.79, *p* = 0.44. For the older WM group a paired t-test revealed that the highest performance of the last session was significantly increased compared to the first session in the numerical complex span task (storage and processing), *t*(9) = -3.16, *p* = 0.01, *d* = -1.00, and in the tower of fame task (relational integration), *t*(9) = -2.75, *p* = 0.02, *d* = -0.87, but not in the figural task switching task (supervision), *t*(9) = 1.79, *p* = 0.11. See Figure B1 for a visualization of the training gains of each task.


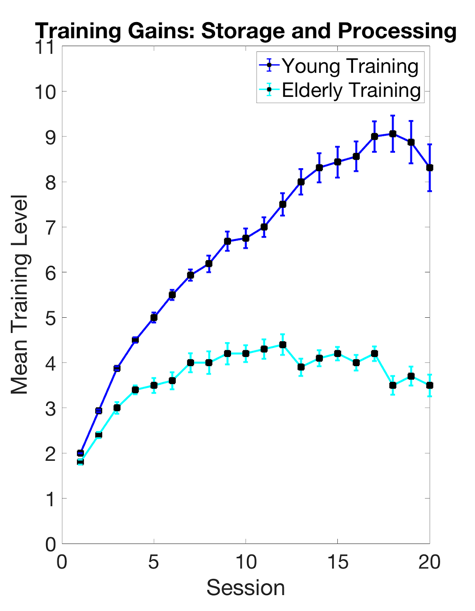

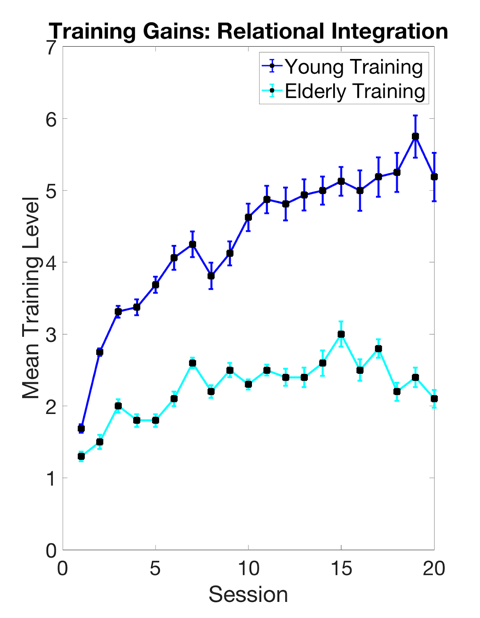

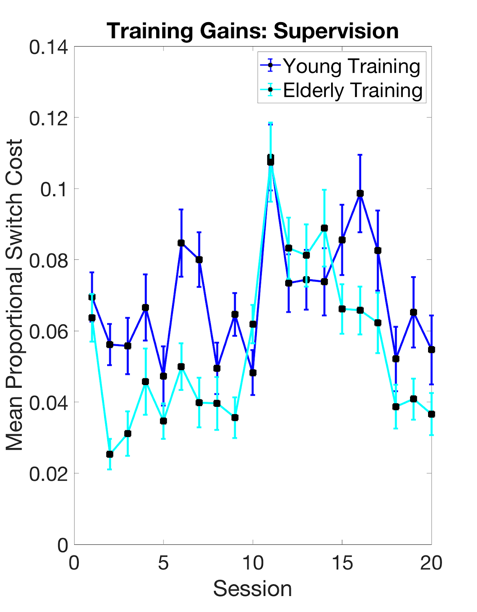


**A       B         C**

**Supplementary Figure B1.** Training gains for the two WM groups (young WM group = blue; older WM group = cyan) over the 20 sessions separately for the three trained tasks: *A:* storage and processing (numerical complex span); *B:* relational integration (tower of fame); *C:* supervision (figural task switching). The x-axis represents the 20 sessions and the y-axis the mean and standard error of the training level in this session for storage and processing, and relational integration, respectively the mean proportional switch costs for supervision.

**Results: Right inferior-longitudinal fasciculus**

The joint generalized linear mixed-effects model for the MD-changes of the right inferior-longitudinal fasciculus revealed an interaction effect of *age × training × time point* on MD changes. To interpret this interaction effect, separate generalized linear mixed-effects models were calculated disjoint for the young and the older group. The results for the separate model for the older group are described in the main publication. The separate model for the young group showed no significant interaction effect of *training* and *time point* as illustrated in the supplementary figure B2 below.


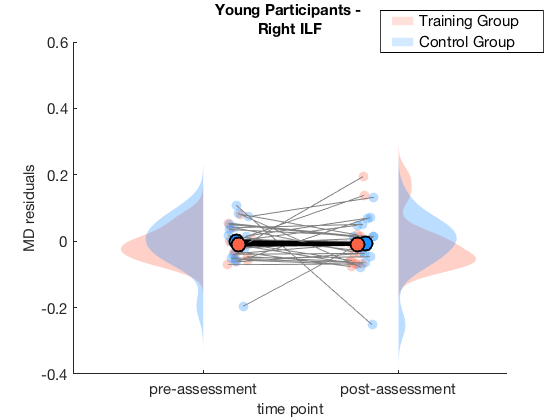


**Supplementary Figure B2.** Averaged MD residuals corrected for CNR, in-scanner head motion, and global MD for the right ILF of the WM group and AC group at pre-assessment and post-assessment of the generalized linear mixed-effects model including only young participants. The generalized linear mixed-effects models showed no significant interaction effect of *training* and *time point*.

**Results: Baseline Differences in MD at Time of Pre-Assessment**

To validate if the observed effects of decrease of MD in right ILF and the right SLF in the WM group compared to the AC group after the training intervention were driven by baseline differences in MD of the given tract, we have conducted a post-hoc analyses using a two-sample t-test. We compared the MD of the tract at time of pre-assessment between the AC and the WM group for each age group separately. The results indicated no significant baseline differences in MD in the right ILF between the AC and the WM group for the young participants, *t*(30) = 0.05, *p* = 0.96, and the older participants, *t*(18) = -1.01 , *p* = 0.33. Similarly for the right SLF, the results revealed no significant baseline differences in MD between the AC and the WM group for the young participants, *t*(30) = -0.95, *p* = 0.35, and the older participants, *t*(18) = -1.66, *p* = 0.11.
